# *MoleculeExperiment* enables consistent infrastructure for molecule-resolved spatial omics data in bioconductor

**DOI:** 10.1093/bioinformatics/btad550

**Published:** 2023-09-12

**Authors:** Bárbara Zita Peters Couto, Nicholas Robertson, Ellis Patrick, Shila Ghazanfar

**Affiliations:** School of Mathematics and Statistics, The University of Sydney, Camperdown, NSW 2006, Australia; Charles Perkins Centre, The University of Sydney, Camperdown, NSW 2006, Australia; Sydney Precision Data Science Centre, The University of Sydney, Camperdown, NSW 2006, Australia; School of Mathematics and Statistics, The University of Sydney, Camperdown, NSW 2006, Australia; Charles Perkins Centre, The University of Sydney, Camperdown, NSW 2006, Australia; Sydney Precision Data Science Centre, The University of Sydney, Camperdown, NSW 2006, Australia; Sydney Laboratory of Data Discovery for Health Limited (D24H), Science Park, Hong Kong SAR, China; School of Mathematics and Statistics, The University of Sydney, Camperdown, NSW 2006, Australia; Charles Perkins Centre, The University of Sydney, Camperdown, NSW 2006, Australia; Sydney Precision Data Science Centre, The University of Sydney, Camperdown, NSW 2006, Australia; Sydney Laboratory of Data Discovery for Health Limited (D24H), Science Park, Hong Kong SAR, China; Centre for Cancer Research, The Westmead Institute for Medical Research, The University of Sydney, Westmead, NSW 2145, Australia; School of Mathematics and Statistics, The University of Sydney, Camperdown, NSW 2006, Australia; Charles Perkins Centre, The University of Sydney, Camperdown, NSW 2006, Australia; Sydney Precision Data Science Centre, The University of Sydney, Camperdown, NSW 2006, Australia

## Abstract

**Motivation:**

Imaging-based spatial transcriptomics (ST) technologies have achieved subcellular resolution, enabling detection of individual molecules in their native tissue context. Data associated with these technologies promise unprecedented opportunity toward understanding cellular and subcellular biology. However, in R/Bioconductor, there is a scarcity of existing computational infrastructure to represent such data, and particularly to summarize and transform it for existing widely adopted computational tools in single-cell transcriptomics analysis, including *SingleCellExperiment* and *SpatialExperiment* (SPE) classes. With the emergence of several commercial offerings of imaging-based ST, there is a pressing need to develop consistent data structure standards for these technologies at the individual molecule-level.

**Results:**

To this end, we have developed *MoleculeExperiment*, an R/Bioconductor package, which (i) stores molecule and cell segmentation boundary information at the molecule-level, (ii) standardizes this molecule-level information across different imaging-based ST technologies, including 10× Genomics’ Xenium, and (iii) streamlines transition from a MoleculeExperiment object to a SpatialExperiment object. Overall, MoleculeExperiment is generally applicable as a data infrastructure class for consistent analysis of molecule-resolved spatial omics data.

**Availability and implementation:**

The *MoleculeExperiment* package is publicly available on Bioconductor at https://bioconductor.org/packages/release/bioc/html/MoleculeExperiment.html. Source code is available on Github at: https://github.com/SydneyBioX/MoleculeExperiment. The vignette for *MoleculeExperiment* can be found at https://bioconductor.org/packages/release/bioc/html/MoleculeExperiment.html.

## 1 Introduction

Spatial omics is a maturing field, especially imaging-based spatial transcriptomics (ST) technologies (Moffitt *et al.* 2022; Wu *et al.* 2022). Since the publication of single-molecule FISH, many imaging-based ST technologies have been developed (Williams *et al.* 2022). While the transcriptome coverage of these technologies is not complete, they enable cellular and even subcellular resolution (Wu *et al.* 2022). In addition, various imaging-based ST technologies have recently started to be commercially shipped, such as 10× Genomics’s Xenium (Janesick *et al.* 2022), NanoString CosMX, and Vizgen MERSCOPE, and thus their use is expected to massively increase in scale. Imaging-based ST has been employed in multiple studies, including investigating the progression from ductal carcinoma in situ to invasive carcinoma (Janesick *et al.* 2022), analyzing the complex immune landscape of the tumor microenvironment in lung tumors (Chen *et al.* 2023), and facilitating the creation of the first comprehensive spatial atlas of the mouse brain (Yao *et al.* 2023). The highly informative cellular and subcellular resolution of imaging-based ST, as well as its increasing commercial availability, motivate the generation of software that helps scientists consistently handle this type of high resolution data.

Recently, *SpatialExperiment* (SPE) was developed as an object class for the study of ST data (Righelli *et al.* 2022). Just like the commonly used SingleCellExperiment class, SPE aims to promote reproducibility of analyses and interoperability of different software on the same data (Amezquita *et al.* 2020; Righelli *et al.* 2022). Moreover, by being a part of the Bioconductor project, these packages are a part of an effort to disseminate open data analysis and promote software maintenance and enhancement in the life sciences (Amezquita *et al.* 2020). However, the SPE package, and more recent extension *SpatialFeatureExperiment* (Moses *et al.* 2023), only allows the storage of gene expression information at the cell or spot level. For molecules that are assigned to cells, their specific locations can be kept via BumpyMatrix assay (https://bioconductor.org/packages/release/bioc/html/BumpyMatrix.html). However, molecules that have not been assigned to a cell, between 5% and 30% of all molecules in a given tissue (Supplementary Table 1), by the cell segmentation method are lost, which is disadvantageous, as these transcripts could yield valuable biological insights (Prabhakaran 2022). For example, performing a region-level differential expression analysis could be more accurate if all detected transcripts are taken into account, even in spaces where transcripts have not been assigned to a cell. However, if one were interested in doing such an analysis on ST data that has been summarized as an SPE object, one would only be able to do this at the cell-level. Therefore, to leverage the molecule resolution of recent technologies, there is a need for a class that avoids premature summarization of ST data, and enables analysis of transcripts in their spatial locations irrespective of cellular compartmentalization.

Beyond the Bioconductor project, there are varied efforts to represent molecule-resolved ST data. The Python package Squidpy (Palla *et al.* 2022) does not handle ST data at the molecule-level, but instead assumes presence of a spatial cell-by-gene counts matrix, while the Seurat R package (Hao *et al.* 2023) has recently been extended to contain molecules as a slot containing an “sp” class object for visualization alongside existing cell-by-gene expression matrices. There have been very recent efforts to build upon image data file formats to include molecule and segmentation information via the OME-NGFF Project (Moore *et al.* 2023), alongside Python package SpatialData (Marconato *et al.* 2023) to read and operate with these data. Overall, there is a collective shift toward representing molecule-resolved ST data beyond the cell-by-gene level, but there is a lack of representation of this currently in R, and particularly in the Bioconductor Project.

In this article, we introduce the MoleculeExperiment class, which represents ST data at the molecule-level. In addition, the MoleculeExperiment class imposes standardized data formats and terminology to avoid the need for manual file conversion and complex analysis scripts of molecule-based ST data. Moreover, the *MoleculeExperiment* package facilitates the transition to a cell-level analysis with the already existing SpatialExperiment class. Here, we enable the application of *MoleculeExperiment* to Xenium (10× Genomics), CosMx (NanoString), and MERSCOPE (Vizgen) data. In summary, the *MoleculeExperiment* package aims to facilitate the downstream analysis of different imaging-based ST data, both at the molecule-level and cellular-level, with the large diversity of data analysis tools in the Bioconductor project.

## 2 Methods

### 2.1 Examination of vendors’ public molecule-resolved spatial transcriptomics data bundles

We examined molecule and boundary data structures from the following technologies: 10× Genomics Xenium, NanoString CosMx, and Vizgen MERSCOPE. We used these vendors’ publicly available output data bundles, in some cases requiring a minimal sign in or form completion. We used these data bundles to inform our readXenium, readCosmx, and readMerscope functions, respectively. In particular, Xenium data correspond to three replicates from fresh frozen mouse brain tissue, accessed online on 8 February 2023; CosMx data correspond to human non-small cell lung cancer, accessed on 27 February 2023; and MERSCOPE data are from human ovarian cancer, accessed on 27 February 2023.

We assessed commonalities in terms of the detected transcripts files as well as cell boundary or segmentation files. No commonalities were found in the cell boundary files across the technologies. Vizgen’s output bundle contains several hdf5 files, Xenium a single csv.gz file, and NanoString has no single file with cell boundaries, but shares the identified cell IDs between the transcript, count matrix, and cell metadata files instead.

### 2.2 Assessing memory requirements of a MoleculeExperiment object

To assess the disk and memory size of molecule data objects, we used the public CosMx data corresponding to the “Lung9_Rep1” sample, with 26 275 891 molecules detected over 900 features. We assessed on disk file sizes for the transcript csv file as made available from the NanoString website, a Gzip compressed csv.gz version of the file, as well as MoleculeExperiment objects exported to disk via readRDS, either including all additional columns or only keeping essential columns. To assess memory sizes, we compared the two MoleculeExperiment objects to a data.frame generated by reading the aforementioned csv file. We quantified file and object sizes using the file.size and object.size functions, respectively, and reported these in megabytes.

### 2.3 Virtual dissection of mouse brain region

To demonstrate the interoperability afforded in *MoleculeExperiment*, we took the tiny subset of the Xenium data and loaded the morphology image tiff into napari (https://napari.org/). Using the shapes tool, we hand-annotated two regions corresponding anatomically to the granule cell layer and molecular layers of the dentate gyrus (Oh *et al.* 2014), and exported this file. We then read this file into R and used the dataframetoMEList() function to assign a new boundaries slot to the MoleculeExperiment object. We then visualized the annotated region and summarized molecule counts in these regions by first adding over counts for cells with centroids within the annotated regions, and second by summarizing directly on molecules using the countMolecules() function. We directly compared the per-gene counts between these regions in scatterplots.

## 3 Results

**Figure 1.**
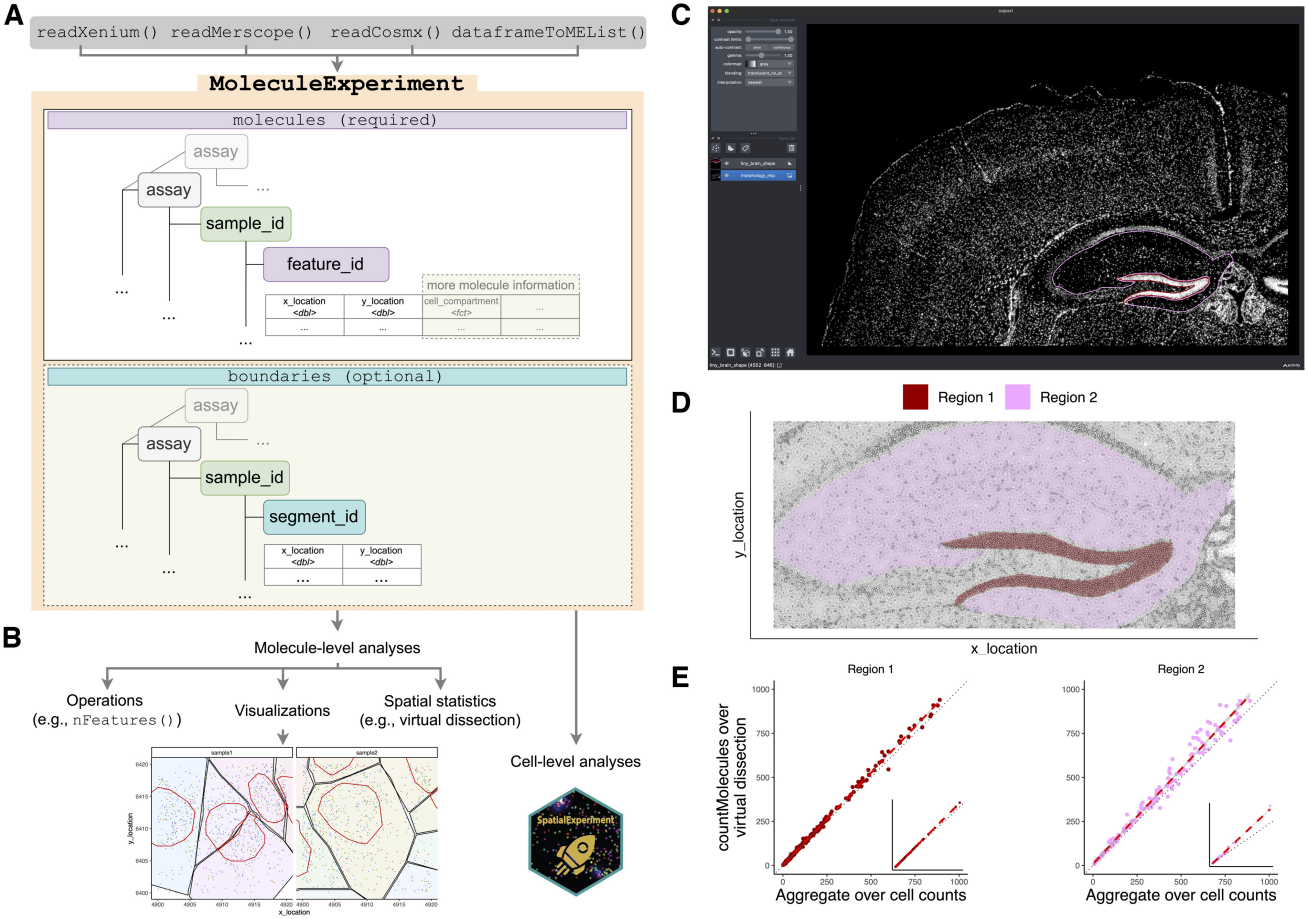
*MoleculeExperiment* aims to facilitate molecule-level and cell-level analysis of data across different vendors of imaging-based ST data. (A) A MoleculeExperiment object has a molecules slot and a boundaries slot, where data format and terminology are standardized (e.g. hierarchical nested list for storage, µm units for the coordinates, and specific column names). The MoleculeExperiment class enables a molecule-centric analysis via class-specific accessor functions. (B) Possible molecule-level downstream analyses include visualizations (e.g. digital in situs), operations (e.g. filtering and counting), and spatial statistics [e.g. Differential gene expression (DGE) by virtual dissection]. In addition, the *MoleculeExperiment* package facilitates transition to a cell-level analysis via the summarization of molecule-level data into a SpatialExperiment object. (C) Screenshot of Xenium mouse brain tiny subset with hand annotation of two regions, in napari. (D) Visualization of annotated regions from *MoleculeExperiment*. (E) Comparison of total molecules detected for each gene in Region 1 (left) and Region 2 (right) showing aggregate over cell counts (x-axes) opposed to countMolecules over virtual dissection region (y-axes). Inset scatterplots are shown over entire dynamic range for all genes. Red dashed lines indicate line of best fit, and gray dotted lines indicate y = x line.

Here, we introduce *MoleculeExperiment*, a core data infrastructure package in R/Bioconductor which enables consistent and reproducible analysis of molecule resolution ST data in the R coding environment (Fig. 1A). The MoleculeExperiment class is an S4 class with one required slot for storing information on molecules and is nested by assay, e.g. for different transcript decoding approaches (Gataric *et al.* 2021; Cisar *et al.* 2023), by sample for datasets with multiple samples and/or images, and by feature_id for different transcripts or molecules. The core information in feature_id is the x_location and y_location of each molecule, but other additional information can be stored here (e.g. molecule-level annotations). The MoleculeExperiment object can contain an additional slot for storing boundaries, which is used for storing various segmentations of the data and is also nested by assay, for different segmentations such as cell bodies, nuclei, or annotated virtual dissections, by sample, and by segment_id for each individual segment (typically a cell).

Due to the large variation in data bundles produced by various vendors of imaging-based ST technologies (Supplementary Figure 1), we have implemented specific functions for reading and standardizing this data into a MoleculeExperiment object. Currently implemented are readXenium(), readCosmx(), and readMerscope(), alongside a technology-agnostic dataFrameToMEList() function. The package provides setter and getter functions, e.g. molecules() and boundaries(), needed to manipulate the object in R. For segmentation information, we enable the reading of both boundaries via dataframeToMEList() as well as segmentation masks via readSegMask() functions.

The hierarchical nested structure of the MoleculeExperiment class avoids redundant storing of information, as opposed to traditional rectangular data storage formats (e.g. csv files). For example, the sample IDs and feature names are not repeated for the millions of molecules corresponding to that sample and feature. As such, *MoleculeExperiment* creates objects that consume less memory than rectangular objects (Supplementary Figure 2). Further, we provide the countMolecules() function that uses parallel computation to summarize the molecule-level data to a typical cell-by-gene representation in a SpatialExperiment object (Righelli *et al.* 2022). Thus, the MoleculeExperiment object not only takes advantage of the molecule resolution of imaging-based ST technologies, but also facilitates the transition from a molecule-level analysis to a cell-level analysis, thereby leveraging the vast capacity of Bioconductor tools designed for single-cell and spatial genomics analysis.

The molecule-centric way in which the MoleculeExperiment object stores data can be used for molecule-level visualizations and statistical analyses (Figure 1B), enabling wrangling of a large proportion of molecule data not overlaid onto existing cell segmentations. One crucial aspect to analysis of molecule-resolved ST is the ability to perform *in silico* virtual dissection and further analysis. We demonstrate the ease of use by virtually dissecting two regions of interest in the napari software (https://napari.org/) from the 10× Genomics Xenium Mouse Brain Tiny Subset data (Figure 1C, Methods) and jointly visualizing in R using *MoleculeExperiment* (Figure 1D). Summarizing over molecules in these regions results in higher recovery of gene counts compared to adding per-cell gene counts, in both dense and sparse tissue regions (Figure 1E).

## 4 Discussion

Here, we have developed MoleculeExperiment, an S4 infrastructure in R/Bioconductor that enables the analysis of imaging-based ST data at the molecule-level, thereby making full use of the molecule resolution that these technologies can achieve. It imposes a standardization of the data such that the data structure, and associated terminology, are consistent across data from diverse vendors of imaging-based ST. This consistent data representation aims to provide a solid foundation for the development of tools for the analysis of molecule-level ST data. We think this is especially important in the current context, where new imaging-based ST technologies and associated analytical methods are constantly being developed (Williams *et al.* 2022; Wu *et al.* 2022). The *MoleculeExperiment* package provides convenience functions to read data from different vendors of imaging-based ST technologies. This aims to simplify the otherwise manual and time-consuming process of in-house data wrangling before data analyses. Finally, the package facilitates summarization into a SpatialExperiment object for downstream cell-level analyses. In this way, it is possible to use already existing Bioconductor packages that work with the SpatialExperiment classes, e.g., *imcRtools* (Windhager *et al.* 2021), *spicyR* (Canete *et al.* 2022), *SPIAT* (Yang *et al.* 2020), and *scHOT* (Ghazanfar *et al.* 2020). Moreover, this means that the molecule-resolved data can be transitioned to other related classes, like *SingleCellExperiment* (Amezquita *et al.* 2020) and its python-equivalent AnnData (Virshup *et al.* 2021). Taken together, by being an S4 Bioconductor class, *MoleculeExperiment* profits from interoperability of downstream software packages, like the SPE (Righelli *et al.* 2022) and *SingleCellExperiment* (Amezquita *et al.* 2020) classes do. Ultimately, the *MoleculeExperiment* package imposes a consistent structure and terminology for imaging-based ST data, with the goal of enabling reproducible downstream molecular- and cellular-level analysis for the user.

Owing to its general nature, *MoleculeExperiment* could be applicable beyond imaging-based ST, and could be relevant to other novel technologies, e.g. lineage barcoding (Frieda *et al.* 2017), and indeed in any situation where discrete molecules are detected in spatial coordinates.

A key advantage of the nested structure of *MoleculeExperiment* is that it stores less redundant information in comparison to rectangular structures (e.g. csv files). Moreover, this hierarchical nested format enables parallelization via BiocParallel (https://bioconductor.org/packages/release/bioc/html/BiocParallel.html) (e.g. within the countMolecules() function). While current molecule-level ST datasets have few replicates, as these technologies increase to larger cohort scales, the need for on-disk representation of non-rectangular data will increase. Tools such as Apache Arrow (https://arrow.apache.org/) or the hierarchical data structure HDF5 (https://bioconductor.org/packages/release/bioc/html/rhdf5.html) may enable further development of on-disk representation of complex non-rectangular data, beyond arrays as used in other Bioconductor packages (Eling *et al.* 2021), that can be incorporated as classes within the *MoleculeExperiment* slots.

In summary, the *MoleculeExperiment* R package standardizes imaging-based ST data at the molecule level across different vendors, and simplifies the steps needed to prepare raw imaging-based ST data, ready for downstream analyses at the cellular- and molecular-level. We hope *MoleculeExperiment* supports the recent and fast-growing spatial omics community.

## Supplementary Material

btad550_Supplementary_Data

## Data Availability

All data used in this study are publicly available. The accession links are reported in the Github repository at https://github.com/SydneyBioX/MoleculeExperiment.
