# Supplementary material for: MoleculeExperiment enables consistent infrastructure for molecule-resolved spatial omics data in bioconductor
Source: Bioinformatics. 2023 Sep 12;39(9):btad550. doi: 10.1093/bioinformatics/btad550 (PMC10504467; doi:10.1093/bioinformatics/btad550)
Supplement: btad550_Supplementary_Data [file btad550_supplementary_data.pdf]

Supplementary Materials

Supplementary Tables

Supplementary Table 1. Number of gene molecules detected across tissues and number assigned to segmented cells.

Sample	Number of total molecules	Number of molecules assigned to segmented cells	Percentage of molecules not assigned to cells
10x Genomics Xenium_V1_FF_Mouse_Brain_MultiSection_1	62,384,369	59,714,127	4.28%
NanoString NSCLC_lung9_rep1	26,184,321	21,516,459	17.8%
Vizgen HumanOvarianCancerPatient2Slice2	31,898,425	22,493,353	29.5%

Supplementary Figures

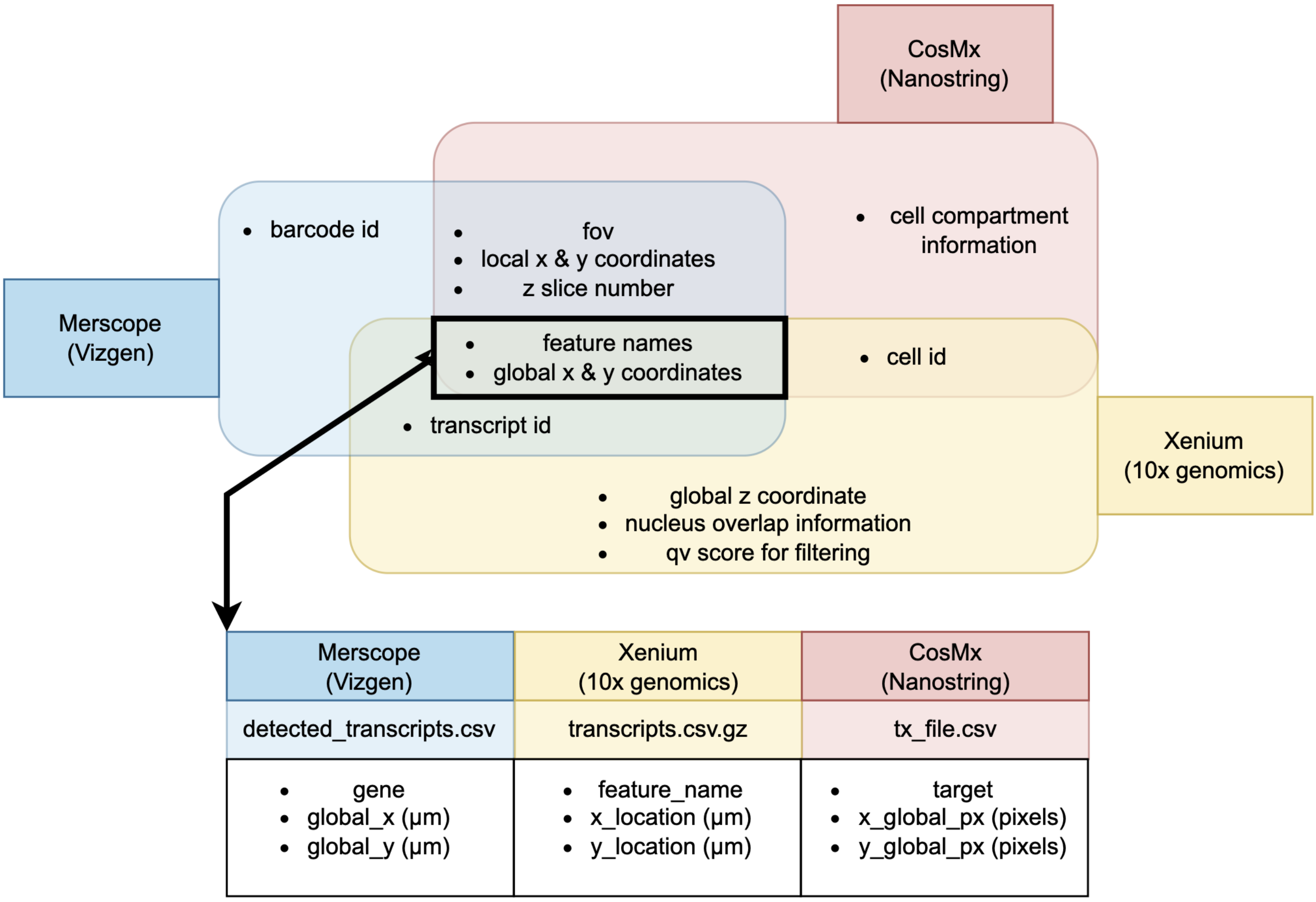

Supplementary Figure 1. Assessment of consistency of data structure among different imaging-based spatial transcriptomics vendors. Venn diagram showing label and type of information in the detected transcript files among technology vendors. Only i) the feature names (including gene names), and ii) the global x and y coordinates of those features are shared. Common information displays different file compression formats, column units and names. The third dimension, z, is given as z-slice numbers in Merscope and CosMx, and as a global z coordinate (in µm) in Xenium.

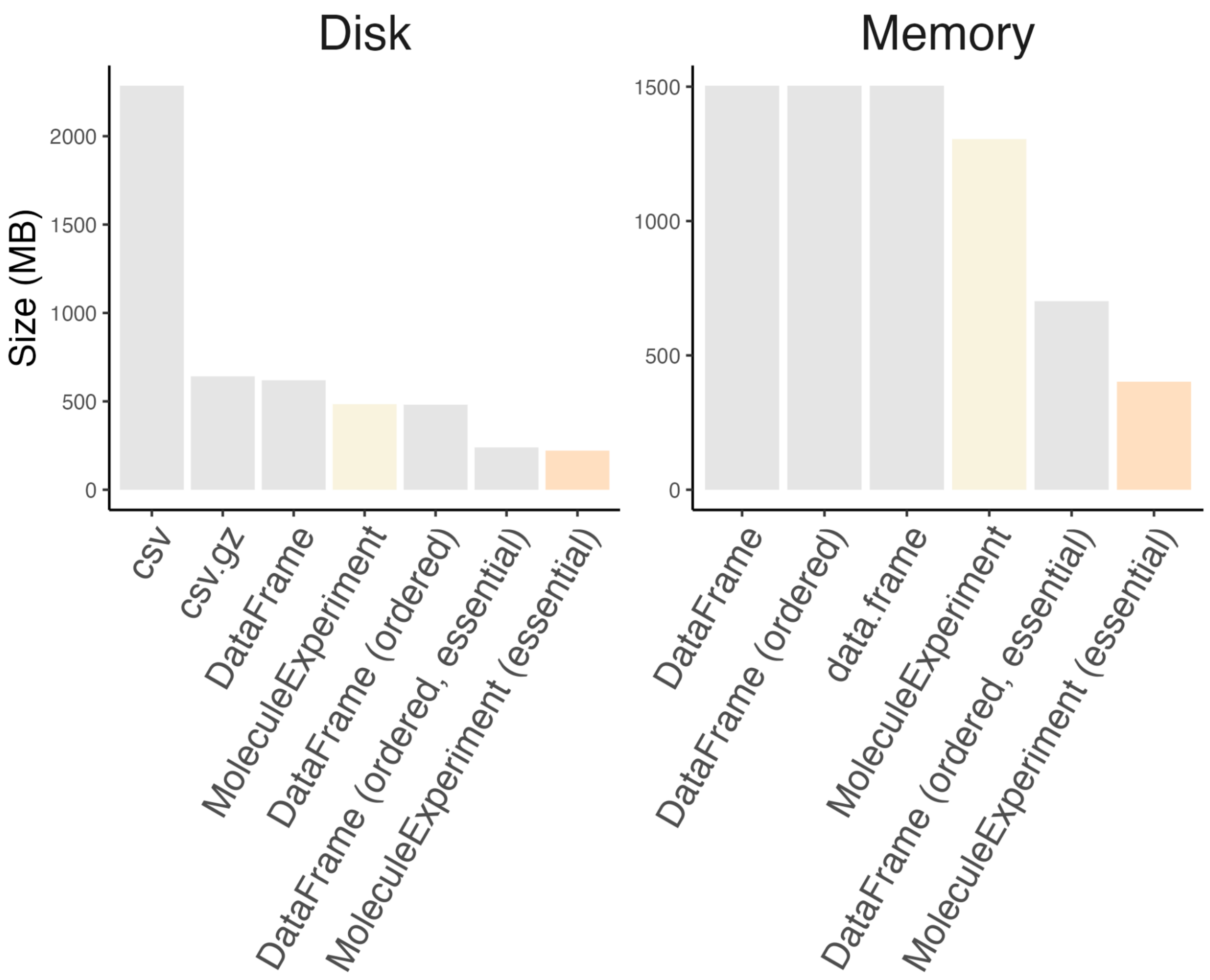

Supplementary Figure 2. Relative memory use of MoleculeExperiment. Barplot displaying memory usage on disk or in memory (MB) of NanoString CosMx molecule-level data for publicly available sample "Lung9_Rep1" when using various infrastructure approaches. On disk storage sizes are shown for csv, gzip compressed (default settings) csv, and Rds files of objects saved using default parametrisation of saveRDS function. In-memory object sizes are shown using the object.size() function.
